# Prognostic significance of white blood cell to platelet ratio in delayed cerebral ischemia and long-term clinical outcome after aneurysmal subarachnoid hemorrhage

**DOI:** 10.3389/fneur.2023.1180178

**Published:** 2023-05-18

**Authors:** Wanwan Zhang, Yifei Wang, Qingqing Zhang, Fandi Hou, Lintao Wang, Zhanqiang Zheng, Yong Guo, Zhongcan Chen, Juha Hernesniemi, Guang Feng, Jianjun Gu

**Affiliations:** ^1^Department of Neurosurgery, Henan University People's Hospital, Henan Provincial People’s Hospital, Zhengzhou, China; ^2^School of Clinical Medicine, Henan University, Kaifeng, China; ^3^Department of Neurosurgery, The Second Affiliated Hospital of Jiaxing University, Jiaxing, China; ^4^Department of Neurosurgery, Henan Provincial People's Hospital, Zhengzhou University People's Hospital, Zhengzhou, China

**Keywords:** aneurysmal subarachnoid hemorrhage (aSAH), delayed cerebral ischemia (DCI), biomarker, white blood cell to platelet ratio (WPR), inflammation, activating platelet, prognosis

## Abstract

**Objectives:**

The ratio of white blood cell to platelet count (WPR) is considered a promising biomarker in some diseases. However, its prediction of delayed cerebral ischemia (DCI) and prognosis after aneurysmal subarachnoid hemorrhage (aSAH) has not been studied. The primary objective of this study was to investigate the predictive value of WPR in DCI after aSAH and its impact on 90-day functional outcome.

**Materials and methods:**

This study retrospectively analyzed the data of blood biochemical parameters in 447 patients with aSAH at early admission. Univariate and multivariate analyses were used to determine the risk factors for DCI. According to multivariate analysis results, a nomogram for predicting DCI is developed and verified by R software. The influence of WPR on 90-day modified Rankin score (mRS) was also analyzed.

**Results:**

Among 447 patients with aSAH, 117 (26.17%) developed DCI during hospitalization. Multivariate logistic regression analysis showed that WPR [OR = 1.236; 95%CI: 1.058–1.444; *p* = 0.007] was an independent risk factor for DCI. The receiver operating characteristic (ROC) curve analysis was used to evaluate the predictive ability of WPR for DCI, and the cut-off value of 5.26 (AUC 0.804, 95% CI: 0.757–0.851, *p* < 0.001). The ROC curve (AUC 0.875, 95% CI: 0.836–0.913, *p* < 0.001) and calibration curve (mean absolute error = 0.017) showed that the nomogram had a good predictive ability for the occurrence of DCI. Finally, we also found that high WPR levels at admission were closely associated with poor prognosis.

**Conclusion:**

WPR level at admission is a novel serum marker for DCI and the poor prognosis after aSAH. A nomogram model containing early WPR will be of great value in predicting DCI after aSAH.

## 1. Introduction

Aneurysmal subarachnoid hemorrhage (aSAH) is a cerebrovascular disease with high disability and mortality rate worldwide. According to previous research, about 1/4 of the patients died within a short time after the onset of the disease. Even with timely medical treatment, about half of the patients were left with severe neurological defects (1). Although the development of modern medical knowledge and technology has effectively reduced the mortality rate and improved the functional prognosis of aSAH patients, some serious complications related to the disease are the main contributors to the poor outcomes. As one of the complications, delayed cerebral ischemia (DCI) plays an important role in the poor prognosis of aSAH. DCI is usually considered to be caused by cerebral vasospasm. According to statistics, about 30% of aSAH patients have ischemic events within 4 to 8 days after the onset of aSAH, which will lead to rapid deterioration of neurological function and eventually severe disability, death, and other adverse events that seriously affect the quality of life of patients (2). However, it remains unclear the pathogenesis of DCI, a complex pathophysiological process involving vascular spasm, thrombosis, inflammation, and other aspects (3). This makes the search for potential predictive DCI clinical features or biomarkers particularly important.

The occurrence of DCI after aSAH has been widely reported regarding age, hydrocephalus, modified Fisher grade (mFisher) and other biomarkers. For example, Plasma Galectin-3, c-reactive protein (CRP), mean platelet volume (MPV), coagulation abnormalities, electrolyte disturbances, and neutrophil/lymphocyte ratio (NLR) are all associated with DCI or poor prognosis of aSAH (4–9). In the latest study, some scholars found that early cerebrospinal fluid lactic acid and glucose levels also have a certain predictive effect in the occurrence of symptomatic DCI (10). However, the relationship between some hematological indicators such as white blood cell and platelet parameters and DCI and functional prognosis after aSAH has not been fully understood. In addition, most of these indicators have previously been independently studied, and the role of their interaction in thrombotic diseases has not been further analyzed. Therefore, the purpose of this retrospective study is to analyze the relationship between white blood cell count and platelet parameters in DCI and functional prognosis after aSAH.

White blood cell/platelet ratio (WPR) is considered as a potential biomarker of vascular inflammation and has been shown to be an independent risk factor for poor prognosis in acute ischemic stroke, cardiovascular risk stratification, severity of liver function, and postoperative infection of renal malignancy (11–16). However, whether WPR is associated with DCI and functional prognosis after aSAH remains unclear. In this study, we analyzed for the first time the relationship between the WPR level at admission, DCI, and prognosis of aSAH patients, so as to facilitate early identification of DCI risk factors and timely intervention measures to improve the quality of life and clinical prognosis of patients.

## 2. Materials and methods

### 2.1. Study population

In this retrospective study, a total of 970 aSAH patients admitted to Henan Provincial People’s Hospital from January 2017 to December 2020 were recruited.

Inclusion criteria are as follows: (1) Patients were diagnosed subarachnoid hemorrhage confirmed by head CT or lumbar puncture, (2) Patients were over 18 years old and aneurysm was responsible for the subarachnoid hemorrhage as confirmed by CT angiography (CTA) and/or digital subtraction angiography (DSA), (3) Embolization or surgical clipping was operated within 48 h after admission, and (4) Hematological specimens were collected at admission or within 48 h of admission for biochemical testing; Exclusion criteria: (1) SAH was caused by non-aneurysmal rupture, (2) Onset time was over 3 days or not clear, (3) aSAH was combined with other cerebrovascular diseases, such as arteriovenous malformations, moyamoya disease, (4) Patients had infectious complications, hematological diseases and autoimmune diseases, (5) Patients received antiplatelet aggregation drugs or other anticoagulant drugs before onset, and (6) Incomplete follow-up data.

**Figure 1:**
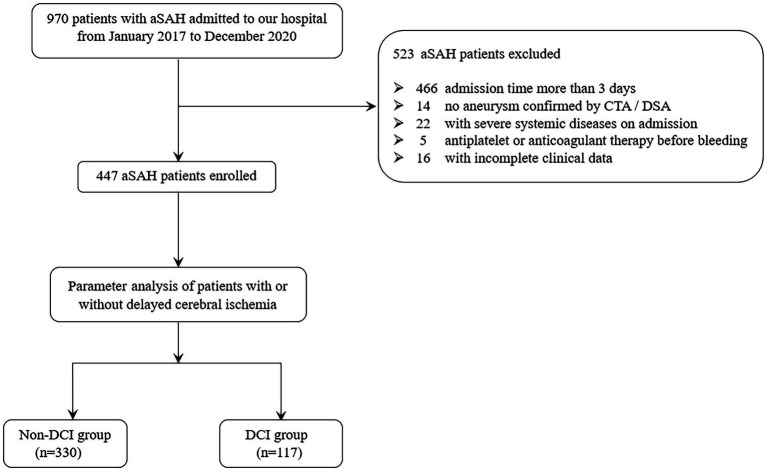
Flow chart of study patients.

The participating patients were followed up 3 months after the onset of the disease, and 447 patients with aSAH were eventually included. The flow chart for inclusion and exclusion criteria is shown in Figure 1. Considering that this study is a retrospective study and does not involve risk to patients themselves, so the written informed consent of patients or their families were not obtained.

### 2.2. Data collection

Patients’ demographic information (gender, age), history (smoking, drinking, diabetes, hypertension, coronary heart disease, stroke history, etc.), and medical records (location/number of aneurysms, hydrocephalus, surgical procedure, etc.) were obtained from patient case management system. Serum biomarkers include white blood cell count, monocyte count, lymphocyte, triglyceride, D-dimer, mean platelet volume, platelet count, platelet-large cell ratio (P-LCR), etc. The WPR in our study was calculated by white blood cell count/platelet count ^*^ 100. Admission CT scans assessed subarachnoid hemorrhage severity by modified Fisher scale and were classified as mild (mFisher1-2) or severe (mFisher3-4). Hunt-Hess grade was used to evaluate the neurological disfunction of patients at admission, which was classified as mild (Hunt-Hess grade 1–3) or severe (Hunt-Hess grade 4–5).

### 2.3. Outcome measurement

The main outcomes included DCI and poor prognosis during hospitalization. According to the previous guidelines, we defined DCI as clinical signs of deterioration caused by newly diagnosed focal nerve function injury, in which patients demonstrated functional changes such as hemiparesis, aphasia, apraxia, hemianopia, neglect, or decreased by at least 2 points on the Glasgow Coma Scale, These conditions last for at least 1 h. And/or a new ischemic or infarct focus in the affected area observed on computed tomography (CT) or magnetic resonance imaging (MRI) (17, 18).The patients were followed up 3 months after the onset of the disease. The modified Rankin Scale (mRS) was used to evaluate the neurological recovery of the patients, which was classified as good prognosis (mRS 0–2) or poor prognosis (mRS 3–6).

### 2.4. Statistical analysis

We used SPSS Statistics 21.0, Origin version 2021 and R version 4.2.1 for statistical analysis and chart production. Firstly, Kolmogorov–Smirnov test was used to analyze the continuous variables to observe whether they fit the normal distribution. The continuous variables that fit the normal distribution were represented by mean ± standard deviation (x ± s), and the difference between groups was compared by two independent samples t test. Continuity variables that did not conform to normal distribution were expressed by median and interquartile range (median, IQR), and comparison of differences between groups was performed by Mann–Whitney U test. Categorical variables were expressed by quantity and composition ratio (n, %), and differences between groups were compared by Pearson’s Chi-squared test. If *p* < 0.05, the parameter difference between the two groups was considered statistically significant. Bivariate correlation analysis was performed using Spearman rank correlation test. The variables with p < 0.05 in the univariate analysis results were included in the multivariate Logistic regression model to determine the independent risk factors associated with DCI. Meaningful factors were analyzed by receiver operating characteristic (ROC) curve. The predictive value of WPR in postoperative DCI in patients with aSAH was analyzed, and the optimal critical value, sensitivity and specificity of WPR in predicting DCI were determined. By constructing a nomogram prediction model, Logistic regression results were visualized to analyze the probability of DCI in different aSAH patients upon admission, and calibration curves were drawn to verify the accuracy and clinical value of this model. In order to analyze the relationship between WPR and prognosis, we divided the low WPR group (≤5.26)/high WPR group (>5.26) according to the optimal threshold value of WPR. Univariate analysis of the prognosis of aSAH patients was performed to compare the differences between the groups.

## 3. Results

This study retrospectively collected 970 patients with aSAH admitted to our hospital, and a total of 447 patients who met the inclusion criteria were analyzed. The average age was 58.74 ± 11.28 years, including 165 male patients (36.91%) and 282 female patients (63.09%).

**Figure 2:**
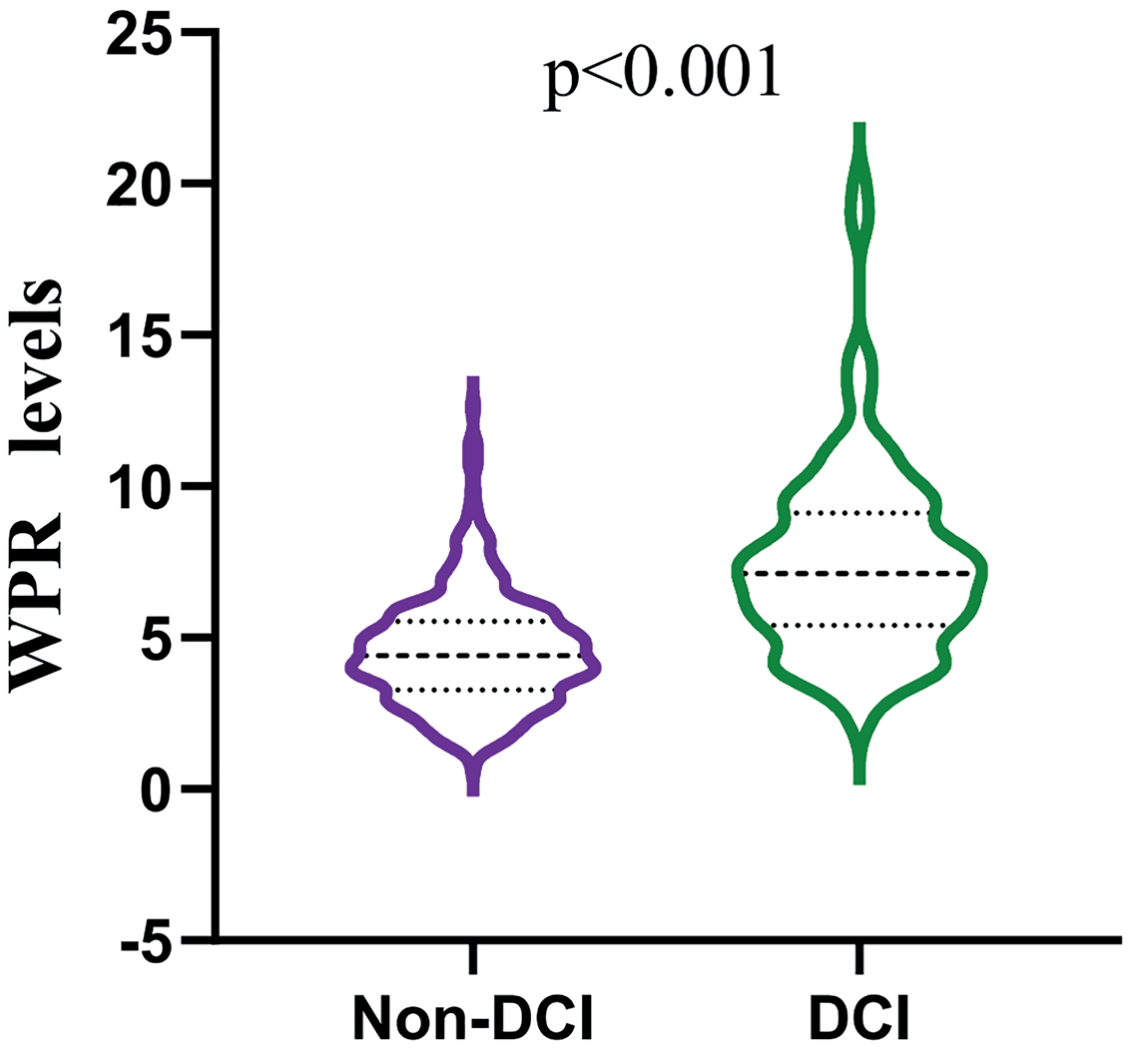
Comparison of white blood patients without and with DCI after aneurysmal subarachnoid hemorrhage. (mean interquartile range 4.40 [3.28–5.54] vs 7.31 [5.40–9.12]; *p* < 0.001).

Our study found that 117 (26.17%) of the 447 patients developed DCI during hospitalization. The baseline characteristics of aSAH patients are shown in Table 1. There were significant differences (*p* < 0.05) between DCI and non-DCI groups in age (60.8 ± 5.95 vs 58.01 ± 11.75, *p* = 0.021), hypertension (68.38% vs 53.94%, *p* = 0.007), hydrocephalus (29.91% vs 19.70%, *p* = 0.023), high Hunt-Hess grade (40.18% vs 13.03%, *p* < 0.001), high mFisher grade (64.96% vs 13.64%, p < 0.001) and clipping (47.01% vs 33.03%, *p* = 0.007). There differences were non-significant in gender, intracranial hematoma, smoking, drinking, diabetes, coronary heart disease, hyperlipidemia, stroke history, location and number of aneurysms (*p* > 0.05). In terms of hematological indicators, the DCI group had significantly higher white blood cells (12.1 (9.35, 15.25) vs 9.73 (7.31, 11.95), *p* < 0.001),triglycerides (1.5 (0.97, 2.04) vs 1.19 (0.91, 1.71), *p* = 0.028), D-dimer (2.13 (0.98, 4.66) vs 1.13 (0.77, 2.13), *p* < 0.001), MPV (11 (10.1, 11.5) vs 10 (9.4, 10.8), *p* < 0.001), p - LCR (34.5 (28.65, 38.5) vs 26.3 (22.4, 32.23), *p* < 0.001), WPR (7.31 (5.4, 9.12) vs 4.4 (3.28, 5.54), *p* < 0.001) and lower PLT (173 (150.5, 193.5) vs 216.5 (189, 262), *p* < 0.001). The level of WPR on admission was closely correlated with DCI (Figure 2).

**Table 1:** Demographic baseline characteristics of patients with delayed cerebral ischemia after aneurysmal subarachnoid hemorrhage.

Variables	Total patients (447)		*p* Value
	Non-DCI group (*n* = 330)	DCI group (*n* = 117)	
Gender			0.531
Female	211 (63.94%)	71 (60.68%)	
Male	119 (36.06%)	46 (39.32%)	
Age, years	58.01 ± 11.75	60.8 ± 5.95	0.021
Intracranial hematoma	44 (13.33%)	16 (13.68%)	0.926
Modified fisher grade			<0.001
GradeI	142 (43.03%)	22 (18.8%)	
GradeII	143 (43.33%)	19 (16.24%)	
GradeIII	17 (5.15%)	23 (19.66%)	
GradeIV	28 (8.49%)	53 (45.3%)	
Hunt-Hess grade			<0.001
GradeI	6 (1.82%)	1 (0.85%)	
GradeII	213 (64.55%)	28 (23.93%)	
GradeIII	68 (20.6%)	41 (35.04%)	
GradeIV	32 (9.7%)	38 (32.48%)	
GradeV	11 (3.33%)	9 (7.7%)	
Cerebral infarction	25 (7.58%)	15 (12.82%)	0.088
Hypertension	178 (53.94%)	80 (68.38%)	0.007
Hydrocephalus	65 (19.70%)	35 (29.91%)	0.023
Smoking	69 (20.91%)	29 (24.79%)	0.384
Drinking	54 (16.36%)	14 (11.97%)	0.255
Diabetes	19 (5.76%)	9 (7.69%)	0.458
Coronary heart disease	27 (8.18%)	15 (11.97%)	0.14
Hyperlipidemia	118 (35.76%)	44 (37.61%)	0.721
Family history	13 (3.94%)	9 (7.69%)	0.107
Aneurysm location (Anterior circulation)	287 (86.97%)	99 (84.62%)	0.524
Number of aneurysms (multiple)	73 (22.12%)	27 (23.08%)	0.831
Aneurysmal treatment			0.007
Endovascular coil	221 (66.97%)	62 (52.99%)	
Surgical clip	109 (33.03%)	55 (47.01%)	
Hematology index			
WBC	9.73 (7.31, 11.95)	12.1 (9.35, 15.25)	<0.001
Neutrophil	9.41 (6.71, 11.95)	9.4 (6.76, 12.48)	0.413
monocyte	0.46 (0.29, 0.63)	0.48 (0.33, 0.74)	0.074
Lymphocytes	0.99 (0.72, 0.34)	0.9 (0.67, 1.29)	0.168
Potassium	3.81 ± 0.47	3.76 ± 0.5	0.393
Phosphate	1.04 (0.89, 1.24)	1.02 (0.9, 1.19)	0.71
Total bilirubin	11.1 (8.68, 14.9)	11.0 (7.55, 15.96)	0.948
Albumin	39.85 (37.0, 42.43)	39.2 (36.3, 42.2)	0.37
Triglyceride	1.19 (0.91, 1.71)	1.5 (0.97, 2.04)	0.028
D-Dimer	1.13 (0.77, 2.13)	2.13 (0.98, 4.66)	<0.001
Plasma fibrinogen	3.22 (2.70, 3.80)	3.27 (2.9, 4.03)	0.244
MPV	10 (9.4, 10.8)	11 (10.1, 11.5)	<0.001
PDW	11.8 (10.68, 13.5)	12.3 (10.8, 14.2)	0.082
PLT	216.5 (189, 262)	173 (150.5, 193.5)	<0.001
P-LCR	26.3 (22.4, 32.23)	34.5 (28.65, 38.5)	<0.001
WPR	4.4 (3.28, 5.54)	7.31 (5.4, 9.12)	<0.001

DCI, delayed cerebral ischemia; WBC, white blood cell; MPV, mean platelet volume; PDW, platelet distribution width; PLT, platelet count; P-LCR, platelet larger cell ratio; WPR, white blood cell to platelet ratio.

Correlation analysis showed that the decrease of lymphocytes, albumin, and platelets and the increase of WPR, white blood cells, neutrophils, D-dimer, and P-LCR were linearly correlated with the increase of the mFisher grading score, which represents the degree of cerebral vasospasm (Table 2).

**Table 2:** Correlation between laboratory markers and modified fisher score.

	Modified fisher score	
	*r*	*p*
WBC	0.427	<0.001
Neutrophil	0.316	<0.001
monocyte	0.109	0.021
Lymphocytes	−0.184	<0.001
Potassium	−0.061	0.2
Phosphate	−0.038	0.418
Total bilirubin	0.015	0.75
Albumin	−0.208	<0.001
Triglyceride	0.148	0.002
D-Dimer	0.322	<0.001
Plasma fibrinogen	0.127	0.007
MPV	0.09	0.058
PDW	0.089	0.06
PLT	−0.222	<0.001
WPR	0.458	<0.001
P-LCR	0.156	<0.001

WBC, white blood cell; MPV, mean platelet volume; PDW, platelet distribution width; PLT, platelet count; P-LCR, platelet larger cell ratio; WPR, white blood cell to platelet ratio.

After excluding other confounding factors, the meaningful index (*p* < 0.05) in the single factor was included in the binary Logistic regression (WBC was excluded due to the high collinearity of WBC and WPR, VIF > 5). The results showed that higher WPR (OR = 1.236; 95%CI: 1.058–1.444; *p* = 0.007) and P-LCR (OR = 1.066; 95%CI: 1.021–1.113; *p* = 0.004) on admission was an independent risk factor for DCI (*p* < 0.05).In addition, the mFisher grading (OR = 2.098; 95%CI: 1.479–2.976; *p* < 0.001), MPV (OR = 1.362; 95%CI: 1.046–1.773; *p* = 0.022), PLT (OR = 0.987; 95%CI: 0.980–0.995; *p* = 0.001) was also independently correlated with DCI, which was consistent with the results reported in previous studies (Table 3).

**Table 3:** Multivariate logistic regression of delayed cerebral ischemia after aneurysmal subarachnoid hemorrhage.

	Odds ratio	95% Confidence interval	*p* value
mFisher grade	2.098	1.479–2.976	<0.001
MPV	1.362	1.046–1.773	0.022
PLT	0.987	0.980–0.995	0.001
WPR	1.236	1.058–1.444	0.007
PLCR	1.066	1.021–1.113	0.004

mFisher, modified Fisher; MPV, mean platelet volume; PLT, platelet count; WPR, white blood cell to platelet ratio; P-LCR, Platelet larger cell ratio.

**Figure 3:**
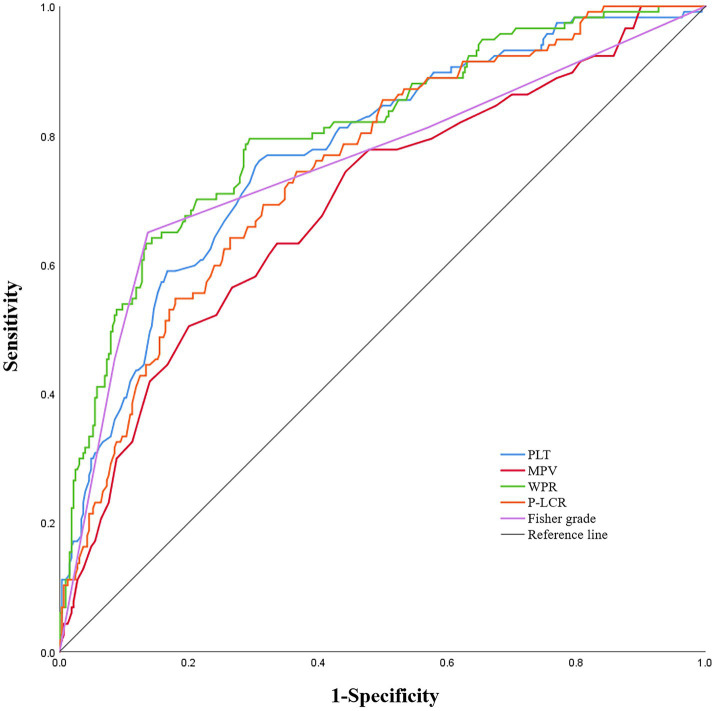
Receiver operating characteristic curve analysis comparing modified Fisher (mFisher) grade, white blood cell to platelet count ratio (WPR), mean platelet volume(MPV), platelet count (PLT)and platelet-large cell ratio(P-LCR)at admission for identifying delayed cerebral ischemia following aneurysmal subarachnoid hemorrhage. The area under the curves of mFisher grade, WPR, MPV, PLT and P-LCR were 0.754 (95%CI: 0. 697–0.811), 0.804 (95%CI: 0. 757–0.851), 0.693 (95%CI: 0. 636–0.750), 0.772 (95%CI: 0. 723–0.822), and 0.749 (95% CI, 0.699–0.799).

In the ROC analysis that using WPR as DCI after aSAH predictor, the optimal cut-off value of the prediction ability of this index was 5.26 (sensitivity 79.5%, specificity 70.6%). In addition, the area under the curve (AUC) of this index was 0.804 (95%CI: 0.757–0.851; *p* < 0.001), area under the curve greater than other risk factors(Figure 3).

**Figure 4:**
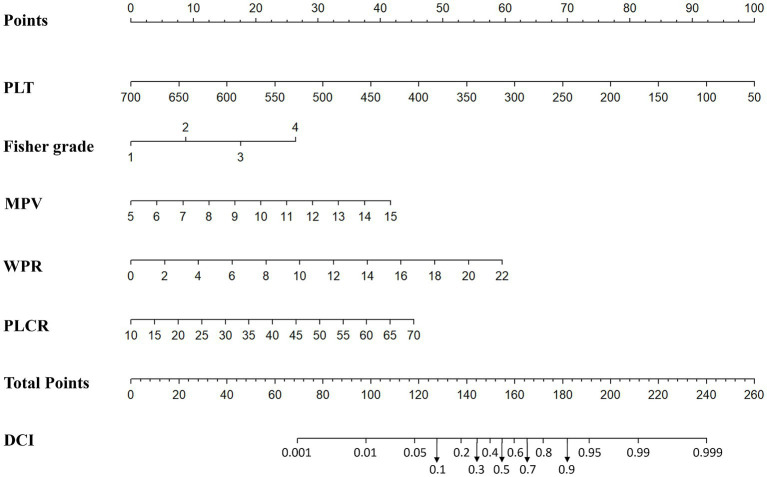
Nomogram prediction of DCI including modified Fisher grade, MPV, PLT, WPR and P-LCR factors.

**Figure 5:**
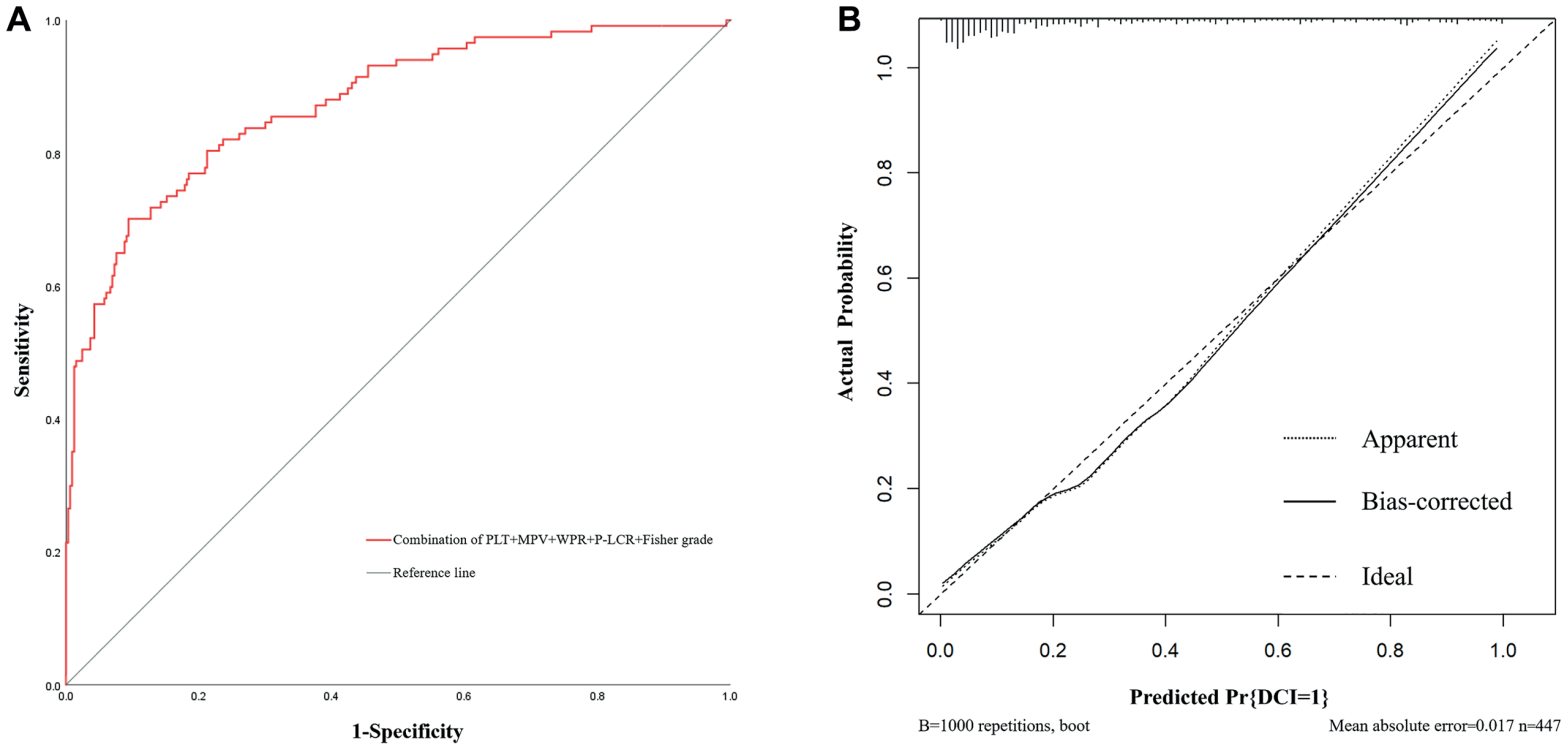
The receiver operating characteristic curve and calibration curves of predictive nomogram for delayed cerebral ischemia. **(A)** The area under the curve is 0.875 (95%CI: 0.836–0.913), indicating that the nomogram has good predictive performance. **(B)** The average absolute error of the calibration curve is 0.017, indicating high accuracy of the nomogram.

In addition, based on the results of multivariate Logistic regression, Nomogram prediction model was constructed by integrating 5 independent risk factors of DCI, Fisher grade, MPV, PLT, P-LCR and WPR (Figure 4). In the early stage of admission, each value level of each influencing factor was scored on the upper scale axis, and each score is added to the total score and projected onto the lower total score scale to estimate the probability of DCI in different patients with different aSAH. The nomogram ROC showed an area under the curve of 0.875 (95%CI: 0.836–0.913; *p* < 0.001), which was better than the predictive efficacy of a single index (Figure 5A).In addition, we used Bootstrap self-sampling method to verify the model internally, and the calibration curve results suggested that the nomogram had high accuracy (Figure 5B).

**Figure 6:**
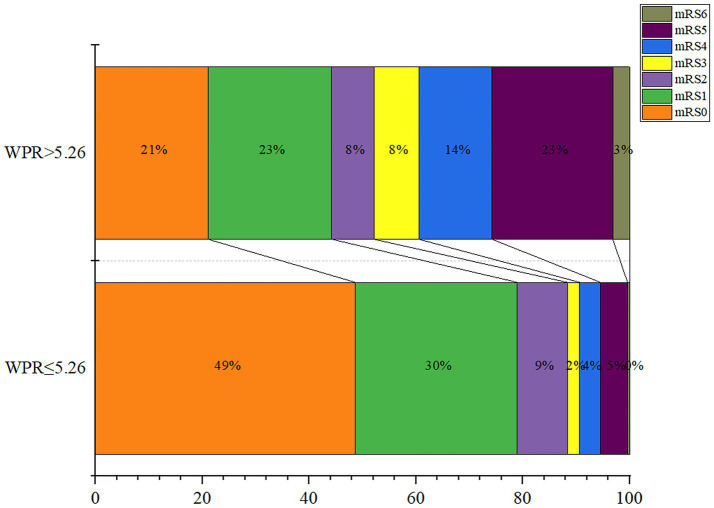
Modified Rankin Scale scores at 3 months in patients with ratio of white blood cell to platelet count (WPR) > 5.26 and WPR ≤ 5.26.

Finally, we divided admission WPR into two groups according to the optimal cut-off value (≤5.26/>5.26), and the statistical results are shown in Table 4. It can be seen that The high WPR group had a higher risk of adverse complications such as intracranial hematoma (*p* = 0.035) and hydrocephalus (*p* < 0.001). High WPR group patients had a higher proportion of previous hypertension (*p* = 0.028) and surgical clipping (*p* = 0.041), and higher Fisher grade (*p* < 0.001), Hunt-Hess grade (*p* < 0.001), DCI incidence (*p* < 0.001), and worse functional prognosis (mRS > 2) (*p* < 0.001).The ordered mRS distribution of patients in the high WPR/low WPR group is shown in Figure 6. Compared with the low WPR group, the high WPR group had a greater proportion of poor prognosis (47.37%vs11.28%; *p* < 0.001), the difference was statistically significant.

**Table 4:** Baseline characteristics of patients dichotomized to the identified WPR threshold (5.26).

Variables	WPR ≤ 5.26 (*n* = 257)	WPR > 5.26 (*n* = 190)	*p* value
Gender			0.711
Female	164 (63.81%)	118 (62.11%)	
Male	93 (36.19%)	72 (37.89%)	
Age, years	120 (46.69%)	97 (51.05%)	0.362
Intracranial hematoma	27 (10.51%)	33 (17.37%)	0.035
Modified fisher grade			<0.001
GradeI-II	232 (90.27%)	94 (49.47%)	
GradeIII-IV	25 (9.73%)	96 (50.53%)	
Hunt-Hess grade			<0.001
GradeI-III	233 (90.66%)	124 (65.26%)	
GradeIV-V	24 (9.34%)	66 (34.74%)	
Cerebral infarction	22 (8.56%)	18 (9.47%)	0.738
Hypertension	137 (53.31%)	121 (63.68%)	0.028
Hydrocephalus	41 (15.95%)	59 (31.05%)	<0.001
Smoking	54 (21.01%)	44 (23.16%)	0.588
Drinking	40 (15.56%)	28 (14.74%)	0.81
Diabetes	13 (5.06%)	15 (7.89%)	0.221
Coronary heart disease	22 (8.56%)	20 (10.53%)	0.481
Hyperlipidemia	91 (35.41%)	71 (37.37%)	0.67
Family history	9 (3.50%)	13 (6.84%)	0.107
Aneurysm location (Anterior circulation)	227 (88.33%)	159 (83.68%)	0.158
Number of aneurysms (multiple)	54 (21.01%)	46 (24.21%)	0.422
Aneurysmal treatment			0.041
Endovascular coil	173 (67.32%)	110 (57.89%)	
Surgical clip	84 (32.68%)	80 (42.11%)	
DCI			<0.001
no	233 (90.66%)	97 (51.05%)	
yes	24 (9.34%)	93 (48.95%)	
mRS score			<0.001
mRS ≤ 2	228 (88.72%)	100 (52.63%)	
mRS >2	29 (11.28%)	90 (47.37%)	

WPR, white blood cell to platelet ratio; DCI, delayed cerebral ischemia; mRS, modified Rankin Scale.

## 4. Discussion

This study is the first to explore the relationship between a novel hematological biomarker WPR and postoperative DCI and neurological outcomes in patients with aSAH. The results illustrated that: (1) The WPR of aSAH patients with DCI was significantly higher than that of the group without DCI, (2) There was a linear correlation between the WPR value and the mFisher grade representing the degree of cerebral vasospasm,(3) After adjusting for some confounding factors, WPR was still a significant factor in DCI in the multivariate analysis, (4) The nomogram prediction model including WPR has a good predictive effect on the occurrence of DCI, and (5) High WPR was closely related to poor prognosis.

aSAH is one of the acute cerebrovascular accidents in neurosurgery caused by blood infiltration into subarachnoid space due to rupture of intracranial aneurysms, which usually leads to a series of neurological dysfunction (19). At present, the common surgical methods mainly include endovascular therapy and craniotomy clipping, which play an important role in the treatment of aSAH patients. In the past three decades, with the rapid development of medical technology, understanding of the pathophysiology, and the intensive care quality of this disease, although the incidence rate has not changed significantly, the survival rate has increased by 17% or even up to 65% compared with the past, which has greatly improved the quality of life of patients (20). However, some complications during the course of the disease still seriously threaten the survival prognosis of patients, among which DCI is the main source of clinical deterioration. In the past, cerebral vasospasm was considered to be the only mechanism for the occurrence of DCI, but later, some scholars found that DCI can occur in the blood supply area of non-cerebral vasospasm, which overturns people’s previous understanding of the mechanism of this disease (21). At the same time, the latest literature indicates that the occurrence of DCI is closely related to the mechanism of arteriolar dysfunction,small thrombosis, cortical diffusion depolarization, and neuroinflammation (22, 23). In view of the fact that the occurrence of DCI is a complex pathophysiological process, more and more hematological biomarkers have become a research hotspot of DCI in aSAH patients in recent years (2, 24, 25). Therefore, identifying accurate and reliable biomarkers is of great importance for monitoring the occurrence and development of DCI and providing timely intervention measures to improve the prognosis of aSAH patients.

The inflammatory process can lead to a series of problems, including accelerating the formation of atherosclerosis, serious tissue damage and promoting platelet aggregation to participate in thrombosis, which plays a crucial role in the development of aSAH disease (26).As one of the important participants in the inflammatory process, white blood cell plays an important role in the inflammatory response and the activation of the coagulation system. Under pro-inflammatory and apoptotic conditions, white blood cell can be induced to express tissue factors containing microvesicles, synthesize and secrete granulozyme, cytokines and other related pro-coagulant substances, and provide coagulation factor activation sites on the surface of leukocytes to regulate coagulation cascades. In addition, activated white blood cells can release powerful platelet activators including cathepsin G and elastase to promote Coagulation cascade reaction and platelet aggregation and activation. Activated platelets interact with white blood cells to form leucocyte - platelet aggregates, which promote the formation of thrombin and microthrombus. The elevation of this aggregate indicator is generally considered to be associated with hypercoagulable thrombotic disease (27, 28). As one of the routine laboratory indicators, white blood cell count is widely studied. NEIL-DWYER et al. first reported that increased WBC count was associated with poor prognosis and cerebral vasospasm after SAH (29). Subsequently, Fawaz Al-Mufti study found that WBC > 12.7 within 72 h after aSAH was closely correlated with DCI, and was higher than the clinical predictive value of modified Fisher score (30). In the latest study, Ieva Buce-Satoba also confirmed that early WBC and CRP count could independently predict the occurrence of secondary cerebrovascular spasm (CV) after aSAH (31). Similarly, in our study, patients with DCI had significantly higher admission WBC counts than those without DCI, which was consistent with previous findings and further confirmed the predictive ability of early WBC count in DCI after aSAH.

Platelet is an important blood component in the human body, which is distributed throughout the vascular system. It can respond to other blood components and signals released by circulation and endothelial cells, and its influence on thrombosis, induction of venous thromboembolism, stroke and myocardial infarction has been widely recognized (32). Previous studies have reported that microthrombus is related with DCI and poor prognosis after aSAH. Shigeharu Suzuki et al. found in an autopsy study on the analysis of microthrombus components in aSAH patients complicated with DCI that microthrombus was mainly composed of aggregated platelet thrombus and fibrin thrombus (33), which fully demonstrated that platelet factors were closely related to the occurrence of DCI. Platelet parameters commonly used in clinical can reflect the function and state of activated platelets. Some scholars have found that some platelet parameters can independently or jointly predict the occurrence of DCI and poor prognosis after aSAH (34–38). In our data, multivariate analysis found that MPV, PLT and P-LCR were independent predictors of DCI. This is due to the increase of platelet activity during acute ischemic attack, which makes the metabolism and function of large platelets more active and releases more large platelets, resulting in the increase of MPV, PDW, PCT and other parameters. At the same time, the consumption of platelets in the circulation increases or consumes more than the production, resulting in the decrease of the number of PLT. This could explain the increase of MPV and P-LCR and the decrease of PLT under DCI.

As a new composite marker composed of white blood cells and platelets, WPR has begun to attract people ‘s attention. In a study on acute ischemic stroke, Chen et al. found that higher WBC and lower PLT count at acute onset were associated with the severity of stroke and poor prognosis at 90 days, suggested that low PWR can be used as an independent predictor of poor prognosis 3 months after intravenous thrombolysis in patients with acute ischemic stroke (13). Basma Abdulhadi et al. pointed out that a higher WPR value on admission would lead to increased mortality during hospitalization after ventricular circulatory support device implantation, and recommended that early assessment of WPR values be used for risk stratification and the development of interventions to reduce mortality (15). In addition, PWR value on admission can also be used to predict adverse complications such as postoperative infection and bleeding in patients with radical nephrectomy. Patients with low PWR have significantly higher risk of adverse complications such as infection and worse prognosis (16). In summary, although the two independent indicators of white blood cell and platelet have been widely studied, the combined effect between these two indicators is still rarely studied in various disease fields, especially WPR has not been reported in aSAH. Therefore, our study analyzed the WPR value of aSAH patients at admission for the first time, and analyzed the predictive effect of this index in DCI and its relationship with prognosis. Our results showed that the initial WPR value at the onset of aSAH patients was closely related to the degree of cerebral vasospasm, which could be used as an independent risk factor for DCI, and also showed good predictive efficacy in poor prognosis.

## 5. Limitation

Nevertheless, our article still has some limitations. First of all, this is a retrospective study, we cannot dynamically observe all the indicators, and some uncontrollable confounding factors may bias the results. Therefore, more prospective clinical trials on the relationship between WPR and DCI and prognosis of aSAH patients are needed to verify the conclusions of our article. Second, this is a single-site study that may have a selection bias, so comparative verification of different patient sources cannot be performed. Finally, with regard to the definition of DCI, for patients who are unconscious or given sedation and lack of CT/MR Imaging, we may exclude some patients who have developed DCI.

## 6. Conclusion

Our results showed that WPR as a novel hematological marker was positively correlated with the mFisher grade representing the degree of cerebral vasospasm. High WPR is an early predictor of DCI in patients with aSAH during hospitalization and has the ability to predict the prognosis of aSAH. The nomogram based on WPR and platelet parameters has a good predictive effect on the occurrence of DCI, which can assist clinical decision-making, early clinical intervention, and reduce mortality and disability.

## Data availability statement

The original contributions presented in the study are included in the article/supplementary material, further inquiries can be directed to the corresponding authors.

## Ethics statement

Ethical approval was not provided for this study on human participants because according to local legislation and institutional requirements, this research did not require ethical review and approval. Written informed consent for participation was not required for this study in accordance with the national legislation and the institutional requirements.

## Author contributions

JG: conceptualization. YG: methodology. WZ, YW, QZ, and LW: data curation. WZ: writing—original draft preparation. JG and GF: writing—review and editing. FH: visualization. ZZ: supervision. ZC: project administration. JH: funding acquisition. All authors contributed to the article and approved the submitted version.

## Funding

This research was supported by Henan Center for Outstanding Overseas Scientists, grant number GZS2022019 and Natural Science Foundation of Henan Province, grant number 222300420412.

## Conflict of interest

The authors declare that the research was conducted in the absence of any commercial or financial relationships that could be construed as a potential conflict of interest.

## Publisher’s note

All claims expressed in this article are solely those of the authors and do not necessarily represent those of their affiliated organizations, or those of the publisher, the editors and the reviewers. Any product that may be evaluated in this article, or claim that may be made by its manufacturer, is not guaranteed or endorsed by the publisher.
